# Supplementary material for: Analysis of PPARγ Signaling Activity in Psoriasis
Source: Int J Mol Sci. 2021 Aug 10;22(16):8603. doi: 10.3390/ijms22168603 (PMC8395241; doi:10.3390/ijms22168603)
Supplement: Supplementary file 1 [file ijms-22-08603-s001.zip › Supplemental materials_Analysis of PPARg signaling activity in psoriasis/Pathway models/Models images and html files/Model of PPARG signaling in psoriasis/285495.html]

H2O2


# Small Molecule H2O2

|  |  |
| --- | --- |
| URN | urn:agi-cas:7722-84-1 |
| Total Entities | 1 |
| Connectivity | 9353 |
| Name | H2O2 |
| Class | Endogenous compound |
| Molecular Weight | 34.014680 |
| XLogP | -0.900000 |

---

|  |  |
| --- | --- |
| ChildConcepts | oxygen |

---

|  |  |
| --- | --- |
| Pathway | Oxidative Stress in Amyotrophic Lateral Sclerosis |
|  | Alzheimer's Disease |
|  | High Level of Homocystine Effects (Methylation Cycle) |
|  | SIRT3 Signaling in Aging |
|  | Protein Oxidation and Aggregation in Aging |
|  | Protein Oxidation and Nitration Products as Disease Biomarkers |
|  | Necrosis |
|  | Eicosanoids in Inflammation |
|  | CD16/CD14 Proinflammatory Monocyte Activation |
|  | Neutrophil Activation via FCGR3B |
|  | Vascular Endothelial Cell Activation by NO |
|  | GFs/TNF -> Ion Channels |
|  | Nociception Expression Targets Signaling |
|  | EDNRA/B -> Vascular Motility |
|  | ADRA1A -> IL6 Production |
|  | ADRA1 -> Prostaglandin Generation |
|  | BDKRB1/2 -> Prostaglandin Generation |
|  | HTR1 -> IL6 Production |
|  | HTR5 -> TNF Production |
|  | HTR1 -> Membrane Transport |
|  | Vitamin B6 (Pyridoxine) Metabolism |
|  | Vitamin A (Retinol) Metabolism and Visual Cycle |
|  | Scavenger Receptor OLR1 in Inflammation Related Endothelial Dysfunction |
|  | Reperfusion Injury in Myocardial ischemia |
|  | Neurons Necrosis Caused by Energy Deficiency |
|  | Oxidative Stress in Vitiligo |
|  | Vitiligo |
|  | beta-Cell Recovery in Diabetes Mellitus |
|  | Hashimoto's Thyroiditis |
|  | Hashimoto's Thyroiditis Triggers |
|  | Iodine Metabolism Related Thyroid Dyshormonogenesis |
|  | Mucin Production in Goblet Airway Epithelial Cells |
|  | CFTR Facilitates Oxidative Stress in Airway Epithelium in CF |
|  | Airway Epithelial Cell Dysfunction |
|  | FXN Role in Friedreich Ataxia Progression |
|  | Glutamate Toxiicity Effects on Striatum Medium Spiny Neurons in Hantington's Disease |
|  | Muscular Dystrophies |
|  | Dystrophin Glycoprotein Complex Signaling in Muscular Dystrophies |
|  | Muscular Dystrophy, Facioscapulohumeral |
|  | Mitochondrial Dysfunction in Alzheimer's Disease |
|  | Estrogen Effects in Mitochondria in Alzheimer Disease |
|  | Mitochondrial Respiratory Chain Dysfunction |
|  | Metals and Amyloid beta Toxicity in Alzheimer's Disease |
|  | Ca2+ Toxicity in Neurons |
|  | Neurotoxin Effects in Parkinson's Disease |
|  | SOD1 Mutation |
|  | Glutamate Mediated Excitotoxicity in Amyotrophic Lateral Sclerosis |
|  | Brain Cell Necrosis in Vascular Dementia |
|  | Androgen Receptor/NKX3-1 Signaling |
|  | Diabetes Induced Cataract |
|  | Age-Related Cataract |
|  | Histidine Metabolism |
|  | Aspartate Metabolism |
|  | Ser/Gly/Thr/Cys Metabolism |
|  | Serine and Glycine Metabolism |
|  | Phenylalanine and Tyrosine Metabolism |
|  | Branched Chain Amino Acids Metabolism |
|  | D-Amino Acid Metabolism |
|  | Urea Cycle and Arginine Metabolism |
|  | Tryptophan Metabolism |
|  | Lysine Metabolsm |
|  | Malonate, Propanoate and beta-Alanine Metabolism |
|  | Glyoxylate and Glycerate Metabolism |
|  | Nicotinate and Nicotinamide Metabolism |
|  | Glutathione Metabolism |
|  | Ascorbate Biosyntesis |
|  | Ethanol Induced Hepatotoxicity |
|  | Doxorubicin Induced Cardiotoxicity |
|  | Cyclosporine Induced Nephrotoxicity |
|  | Cocaine Induced Hepatotoxicity |
|  | ROS Metabolism |
|  | Asbestosis- and Silicosis-Associated Inflammation Provokes Cancer Development |
|  | Oxidized LDL/OLR1 Activates Cell Proliferation in Cancer |
|  | VEGFA Dependent Angiogenesis in Cancer |
|  | Omega-6-Fatty Acid Metabolism |
|  | Omega-3-Fatty Acid Metabolism |
|  | Purine Metabolism |
|  | AGT -> TP53 Expression Targets |
|  | AGT -> CREB Expression Targets |
|  | AGT -> ELK/SRF Expression Targets |
|  | ROS in Neutrophil Mediated Cell Damage |
|  | ROS in Triggering Vascular Inflammation |
|  | Glutamate Mediated Neurotoxicity |
|  | Mitochondrial Damage Induced by Hypoxia |
|  | ROS and RNS in Vasoconstriction and Vasodilation Regulation |
|  | Ca2+ Cytosolic Overload |
|  | ROS in Angiotensin Mediated Cardiovascular Remodeling and Hypertrophy |
|  | Sulfur Metabolism |
|  | Caffeine Metabolism |
|  | Model of PPARG signaling in psoriasis |
|  | 2\_Diabetes-induced cataract |
|  | 1\_Age-related cataract |
|  | 2\_Oxidized LDL causes endothelial dysfunction in atherosclerosis |
|  | 1\_Endothelial cells stimulate vasoconstriction and thrombosis in pulmonary hypertension |
|  | 3\_2\_Regulation of airway surface liquid (ASL) thickness |
|  | 3\_1\_Mucus accumulation in asthma |
|  | 4\_Mucin hyperproduction in goblet and mucous cells in COPD |
|  | 2\_Hyperglycemia and hyperlipidemia trigger beta-cell apoptosis |
|  | 3\_1\_Beta-cell proliferation and compensation: proliferation |
|  | 3\_2\_Beta-cell proliferation and compensation: neogenesis/dedifferentiation |
|  | 2\_Ion channel CFTR failure in cystic fibrosis airway epithelium (class III-IV mutations) |
|  | 1\_Glutamate and calcium excitotoxicity of motor neurons in ALS |
|  | 2\_Mutations in SOD1 and associated proteins cause motor neuron death in familial ALS |
|  | 1\_Decreased secretion of thyroid hormones |
|  | 1\_cd\_Amyloid-beta in Alzheimer's disease |

---

|  |  |
| --- | --- |
| MedScan ID | 1092767 |

---

|  |  |
| --- | --- |
| Alias | oxzone |
|  | 66554-50-5 |
|  | 8007-30-5 |
|  | hydrogen peroxide hydroperoxide |
|  | UN 2015 |
|  | 60593-56-8 |
|  | Glycozone |
|  | HSDB 547 |
|  | truzone |
|  | Perhydrol |
|  | Pyrozone |
|  | 7722-84-1 |
|  | Kastone |
|  | Hioxy |
|  | Waterstofperoxyde |
|  | UN 2014 |
|  | Superoxol |
|  | hydrozone |
|  | mirasept |
|  | Peroxaan |
|  | Elawox |
|  | peroxol |
|  | peroxal |
|  | lensan a |
|  | Wasserstoffperoxid |
|  | hydrogen peroxide (aqueous) |
|  | Opalescence Xtra |
|  | eskata |
|  | dioxogen |
|  | CCRIS 1060 |
|  | 218625-72-0 |
|  | H2O2 |
|  | dioxidaniumyl |
|  | Peroxan |
|  | Caswell No. 486AAA |
|  | 37355-84-3 |
|  | Hydrogen dioxide |
|  | EINECS 231-765-0 |
|  | peroxyl |
|  | H(2)O(2) |
|  | NSC 19892 |
|  | UN 2984 |
|  | pegasyl |
|  | Perone |
|  | hydrogen peroxide |
|  | Puresept |
|  | Hioxyl |
|  | Hydrogen peroxide (H2O2) |
|  | Oxogen |
|  | Genoxide |
|  | Inhibine |
|  | Lensept |
|  | EPA Pesticide Chemical Code 000595 |
|  | Dihydrogen dioxide |
|  | Interox |
|  | HOOH |
|  | Oxydol |
|  | Peroxide |
|  | oxysept |
|  | [18O2]hydroperoxide |
|  | Hydroperoxide |
|  | Hydrogen dioxide solution |

---

|  |  |
| --- | --- |
| CAS ID | 7722-84-1 |
|  | 218625-72-0 |
|  | 37355-84-3 |
|  | 66554-50-5 |
|  | 8007-30-5 |

---

|  |  |
| --- | --- |
| Reaxys ID | 16069320 |
|  | 16069321 |
|  | 16250093 |
|  | 16250207 |
|  | 2093062 |
|  | 3587191 |

---

|  |  |
| --- | --- |
| ChEBI ID | 16240 |
|  | 30492 |

---

|  |  |
| --- | --- |
| PharmaPendium ID | Hydrogen Peroxide |

---

|  |  |
| --- | --- |
| HMDB ID | HMDB03125 |
|  | HMDB06491 |

---

|  |  |
| --- | --- |
| KEGG ID | C00027 |

---

|  |  |
| --- | --- |
| InChIKey | MHAJPDPJQMAIIY-UHFFFAOYSA-N |

---

|  |  |
| --- | --- |
| Molecular Formula | H2O2 |

---

|  |  |
| --- | --- |
| PubChem SID | 134989344 |
|  | 166916 |

---

|  |  |
| --- | --- |
| PubChem CID | 784 |

---

|  |  |
| --- | --- |
| XLogP-AA | -0.9 |

---

|  |  |
| --- | --- |
| IUPAC Name | hydrogen peroxide |

---

|  |  |
| --- | --- |
| PharmaPendium Name | Hydrogen Peroxide |

---
